# Epidemiology of Hypertension in a Typical State-Level Poverty-Stricken County in China and Evaluation of a Whole Population Health Prevention Project Intervention

**DOI:** 10.1155/2019/4634823

**Published:** 2019-12-11

**Authors:** Zhengye Li, Xingyu Liu, Zhongan Zhang, Li Huang, Qing Zhong, Renlin He, Pei Chen, Ailin Li, Jun Liang, Jianbo Lei

**Affiliations:** ^1^People's Hospital of Xuyong County, Luzhou, Sichuan Province, China; ^2^Affiliated Hospital of Stomatology, Southwest Medical University, Luzhou, Sichuan Province, China; ^3^Performance Management Department, Qingdao Central Hospital, Qingdao, Shandong Province, China; ^4^Teaching and Research Lab of Sociology, Southwest Medical University, Luzhou, Sichuan Province, China; ^5^IT Center, Second Affiliated Hospital, School of Medicine, Zhejiang University, Hangzhou, Zhejiang Province, China; ^6^School of Medical Informatics and Engineering, Southwest Medical University, Luzhou, Sichuan Province, China; ^7^Center for Medical Informatics, Peking University, Beijing, China; ^8^Institute of Medical Technology, Health Science Center, Peking University, Beijing, China

## Abstract

**Background:**

In China, there were 584 state-level, poverty-stricken counties until January 2019. The central government has invested a large amount of funds and preferential policies to alleviate poverty in these areas. The Whole Population Preventive Healthcare Pilot Project (WPPHCPP) aims to explore the use of limited funds to achieve healthy poverty alleviation through free regular physical examinations and comprehensive health management for the entire population in impoverished regions.

**Objective:**

By demonstrating the prevalence of hypertension in populations of poverty-stricken counties in Western China and evaluating health management outcomes after implementing the WPPHCPP, we can provide a foundation for the future development and promotion of improved public health.

**Subjects and Methods:**

Through the WPPHCPP, the entire population in the pilot area was required to undergo free physical examinations. The examinations screened for hypertension revealed the epidemiology of adult hypertension. Based on blood pressure levels and risk factor exposures, risk classifications for hypertensive patients were performed. Corresponding intervention and management strategies for different risk levels were provided by a joint management team consisting of family physicians from three different levels of local medical institutions (village, town, and county). Healthcare management outcomes including awareness, treatment, and hypertension disease rates were compared between the period before and after the intervention and management.

**Results:**

By the end of 2017, among the 452,200 permanent residents in the region, 285,458 adults had completed the physical examination. The prevalence of hypertension was 18.5%, which was lower than the national average of rural areas (28.8%). The prevalence of hypertension in men (18.7%) was slightly higher than that of women (18.3%). The prevalence of hypertension increases with age; for people aged >65 years, it was 39.2%. There were 15,074 newly discovered hypertensive patients in the WPPHCPP, accounting for 29.6% of the total hypertensive population in the region. Regarding the management outcomes, the rates of management and standardized management of hypertension increased each year between 2015 and 2017. Although the rate of disease control management decreased slightly, the overall level of management remained significant. The awareness and treatment rates of hypertension also increased over the years and peaked at 95.0% and 94.9%, respectively, in 2017. The disease control rate was 45.6% in 2016, which was the highest among the years assessed. All the above parameters were better than the national average of rural areas. From 2015 to 2017, the number of people with high-risk factors for hypertension and percentage of high-risk patients decreased from 33,064 to 26,982 and 27.4% to 24.6%, respectively. The percentage of the population exposed to cigarettes and alcohol decreased from 30.6% to 27.2% and 25.1% to 22.0%, respectively. The number of deaths due to hypertensive cardiovascular or cerebrovascular diseases decreased each year and was 275 (39.55/100,000 people) in 2017, which was the lowest rate measured. The annual growth of cardiovascular or cerebrovascular diseases remained negative.

**Conclusions:**

The overall prevalence of hypertension in the studied area was lower than the national average of rural areas. The health management model of “government-led joint efforts of three levels of medical institutions (village, town, and county) with active participation of local village communities” improved the management outcomes of hypertensive patients and fulfilled the latest advocacy of the prevention and control of chronic diseases by the United Nations. This model can be considered an effective model for healthcare management practice in similar situations.

## 1. Introduction

Hypertension is a common cardiovascular symptom and an important independent risk factor for cardiovascular and cerebrovascular diseases, such as coronary heart disease and stroke [1–4]. Prevention and treatment of hypertension is a global challenge in public health [5–8]. In China, hypertension is the leading cause of premature death [9–11]. In 2009, health management for hypertensive patients was added to the National Basic Public Health Services (NBPHS) project, but the outcomes have been unsatisfactory [12]. The Chinese government has conducted a total of six national sampling surveys on hypertension. According to the latest national sampling survey, the nationwide prevalence of hypertension among residents aged ≥18 was 27.9% as of 2015. Compared to the prevalence of 25.1% in 2012, the prevalence of hypertension is still increasing. Less than 50% of patients diagnosed with hypertension received treatment, and the rate of hypertension control was only 37.5% [13]. The rates of awareness, treatment, and control of adult hypertension in rural areas were all lower than those in urban regions during the same period [14]. Therefore, the prevention and control of hypertension is still a significant challenge faced by the Chinese government, specifically in impoverished areas where hypertensive patients are susceptible to poverty-induced illness and return to poverty due to illness, which further impoverishes these regions.

Hypertension and its complications are the major causes of disability and mortality, leading to approximately 10 million global deaths in 2013 [15]. Atrial fibrillation is one of the most common complications of chronic hypertension. Studies have shown that the incidence of atrial fibrillation in hypertensive patients increases by 1.7 times. Hypertension and atrial fibrillation often coexist, and one in six patients with atrial fibrillation was attributed to hypertension [16]. Therefore, the most important measure to prevent atrial fibrillation is to actively reduce blood pressure and to avoid ventricular hypertrophy and atrial enlargement. Although the prevalence of hypertension is similar in urban and rural areas, the awareness rate, treatment rate, and control rate of hypertension in rural areas are far lower than those in urban areas, which poses greater challenges in controlling hypertension and preventing atrial fibrillation in rural areas [17].

Governments of many nations have realized that the prevention and control of chronic diseases such as hypertension is not only a grave public health concern but also a serious problem for political, economic, and social development [17, 18]. The Chinese government is committed to fighting and alleviating poverty-impoverished areas and even listed this plan in the national program of “Healthy China 2030” [19]. Until January, 2019, China had 584 state-level, poverty-stricken counties, referring to counties with a per capita annual income <400 yuan ($57) in the 1992 survey, such as Xuyong County, Luzhou, Sichuan [20]. Xuyong County is located in the southern margin of the Sichuan Basin, bordering Yunnan and Guizhou. The county covers an area of 2,977 square kilometers with 25 townships, 230 administrative villages, and 35 communities. The registered population is 729,300, and the resident population is 452,200. There are 30 minority ethnic groups with a total population of 78,000, including the Miao, Yi, Hui, and Man. There are 90 poverty-stricken villages in the county and 97,290 people living in poverty, of which 9,670—or 40.9% of impoverished households—are poor due to illness. To solve this problem, Xuyong County has implemented an innovative pilot project of preventive healthcare (the Whole Population Preventive Healthcare Pilot Project (WPPHCPP)) [21] for all villagers since October 2014. The WPPHCPP performs the following three basic components of healthcare management [22]: free physical examination for the resident population, establishment of health records, and intervention, management, and prevention of health risks. The WPPHCPP uses various approaches by following the implementation model of government-led health management with joint forces of medical institutions from three administrative levels of local government agencies (county, town, and village) and participation from village and community leaders. The program has achieved a series of successful results and attracted much attention from all over the country. However, a full evaluation of this project's outcomes has not been performed. In this study, we used hypertension as a representative example and investigated the prevalence of hypertension in Xuyong County, accurately describing the disease status in the total population of Xuyong for the first time. Additionally, we implemented a special model of the universal preventive healthcare program WPPHCPP for hypertensive patients in rural China and assessed the effectiveness of such a healthcare intervention and management program.

## 2. Subjects and Methods

The special program, special fund, and pilot project “WPPHCPP” is established by the government with the goal of poverty alleviation by investigating effective measures to prevent and control the health of the whole population in pilot poor areas.

### 2.1. Sources of Information

The research subjects were composed of individuals who underwent physical examinations provided by the WPPHCPP in Xuyong County that started from October 1, 2014. After each resident has completed the physical examination in various health spots, the medical examination report was directly entered into a web-based information management system by doctors or nurses. The so-called grassroots health and medical information system is developed and deployed by Sichuan Provincial Department of Health and is used by the whole province. Data from October 1, 2014, to October 31, 2017, were exported for analysis. The exported data included the following features: health records of adult residents, hypertension risk information, parameters related to hypertension during physical examination, and follow-up records of blood pressure levels. The data involving deaths due to hypertensive cardiovascular and cerebrovascular diseases originated from the death monitoring system of Xuyong County from 2014 to 2017, and the death index was calculated based on the registered number of households provided by the county government.

### 2.2. Screening and Diagnostic Criteria for Hypertension [23, 24]

Patients screened for hypertension in the physical examination were classified into two groups: group 1 had disease known before the WPPHCPP, as determined by the past medical history, and group 2 was composed of patients newly diagnosed with hypertension by the WPPHCPP. The diagnostic criterion was as follows: the person's blood pressure during checkup met the diagnostic criteria for hypertension (systolic blood pressure ≥140 mmHg and/or diastolic blood pressure ≥90 mmHg) but without a prior history of hypertension. According to the blood pressure measurement guide of China [25], a second measurement in a relatively quiet room (the retest room) is required; if the blood pressure still meets the standard high blood pressure, then another two measurements should be performed within 4 weeks. If the patient meets the diagnostic criteria for hypertension 4 times, hypertension is diagnosed. Additionally, if the patient's blood pressure is critically high (systolic blood pressure ≥180 mmHg and/or diastolic blood pressure ≥110 mmHg) during the physical examination and remains critically high in the second measurement in the retest room where other disturbances are excluded, the patient will also be diagnosed with hypertension.

### 2.3. Evaluation Parameters for Management Outcomes

The data from the physical examination and related variables from the death monitoring system were imported into Statistical Package for the Social Sciences version 24.0. Parameters related to hypertension were calculated, illustrated, and compared (as listed below). Among them, parameters (6) and (7) were compared between the first and the last physical examination for hypertensive patients who participated in at least two physical examinations; parameters (3), (4), (5), and (8) were analyzed as yearly statistics. The parameters are as follows:

1. General patient demographics including the number of patients and ratios of genders and ages.
2. The number and prevalence of hypertensive patients: the number of patients refers to the total number of hypertensive patients including those who had a history of hypertension and those who were newly diagnosed with hypertension during physical examinations. The prevalence rate refers to the percentage of hypertensive patients among the total number of adults who underwent the physical examination.
3. Rate of newly detected hypertension: it refers to the proportion of newly diagnosed hypertensive patients to the total number of hypertensive patients.
4. Outcome parameters for health management of hypertensive patients [26]: the rate of standardized management of hypertensive patients = the number of hypertensive patients management according to standardized requirements/the number of hypertensive patients managed during the year ×100%; the rate of blood pressure control in the managed population = the number of managed patients achieving normal blood pressure at the most recent follow-up of the year/the number of managed hypertensive patients of the year ×100%. All data were collected from the follow-up records.
5. “Three rates” for hypertension prevention and treatment [27]: the awareness rate of hypertension = the number of people who know about their own hypertension/the total number of hypertensive patients ×100%; the hypertension treatment rate = the number of hypertensive patients taking blood pressure medication/the total number of hypertensive patients ×100%; the rate of hypertension control = the number of people who reached normal blood pressure with the help of hypertension control/the total number of people with hypertension ×100%. The awareness rates and rates of disease control were extracted from the information collected at the physical examination, and the treatment rates were obtained from the follow-up records.
6. Risk classification of hypertensive patients [27]: *Guidelines for the Management of Hypertension in the Grassroots in China* classify hypertension into 3 levels: low risk (Grade 1), intermediate risk (Grade 2), and high risk (Grade 3), based on blood pressure levels and exposures to risk factors.  Table 1 presents the specific classification criteria.
7. Factors affecting cardiovascular prognosis in hypertensive patients [25] include cigarette smoking, obesity (body mass index ≥28 kg/m^2^), abdominal obesity (waist circumference: men ≥90 cm, women ≥85 cm), impaired glucose tolerance (fasting blood glucose level 6.1–6.9 mmol/L), diabetes (fasting blood glucose level ≥7.0 mmol/L), blood lipids (total cholesterol (TC) level ≥5.7 mmol/L, low-density lipoprotein cholesterol (LDL-C) level >3.3 mmol/L, and high-density lipoprotein cholesterol level <1.0 mmol/L), and serum creatinine level (men ≥133 *μ*mol/L, women ≥124 *μ*mol/L). The frequency and composition proportion of each risk factor were calculated.
8. Death index: we mainly analyzed death caused by hypertensive cardiovascular and cerebrovascular diseases (including coronary heart diseases, myocardial infarction, stroke, and other diseases). The mortality rate = the number of deaths/the number of registered individuals ×(1/100,000); annual growth = (current year mortality−previous year mortality)/previous year mortality ×100%.

## 3. Results

### 3.1. General Information on the Research Subjects

As of the end of October 2017, 285,458 adults had participated in the WPPHCPP free checkups, accounting for >90% of the resident population aged ≥18 years in Xuyong County. Among them, there were 135,564 (47.5%) men and 149,827 (52.5%) women with 2 missing values and gender of 65 people unknown. The average age of the patients undergoing physical examination was 51.42 ± 16.85 years. Of this group, 68,156 people were aged 65 years and above and accounted for 23.9% of the total group, suggesting the existence of a large aging population (Table 2).

### 3.2. The Prevalence of Hypertension among the Subject Population

#### 3.2.1. Adult Hypertension

By the end of 2017, the prevalence of adult hypertension in the region was 18.5%, and the prevalence in men (18.7%) was slightly higher than that in women (18.3%). The prevalence rates increased with age, and 39.2% of individuals over 65 years had hypertension (Table 3).

### 3.3. Management Outcomes for Adult Hypertensive Patients

#### 3.3.1. Newly Detected Hypertension

From 2015 to 2017, a total of 15,074 hypertensive patients were diagnosed through the WPPHCPP, accounting for 29.6% of all hypertensive patients. The number of newly diagnosed men (7,869, 32.1%) was higher than that of newly diagnosed women (7,202, 27.4%). The highest number of adults newly diagnosed with hypertension was observed in 2015 for both different genders and individual age ranges, followed by 2016. The specific numbers of diagnosed adults and their percentages are shown in Table 4.

### 3.4. Outcome Parameters for Health Management of Hypertensive Patients

During the WPPHCPP implementation, the rates of management and standardized management increased annually and peaked at 88.4% and 55.9%, respectively, in 2017, whereas the disease control rate in the managed population continued to decrease, although it was 67.8% in 2017 which was higher than 65%, a government required standard (Table 5). There is a special phenomenon that deserves further clarification. Usually, with the increase of the management rate and standardized management rate, the disease control rate should be improved, but in fact, it has declined. The reasons are as follows: the management rate and the standardized management rate are relatively easy to improve because they mainly involve whether to intervene or not, regardless of the final outcomes. But the standardized control depends on whether the blood pressure, the most important outcome indicator, is controlled. The actual situation is that the blood pressure control is not easy. Drug treatment is very important, and grassroots doctors need a longer time to master the method of drug treatment for effective outcomes. Another important reason is that, after the project has been started, comprehensive quality control such as strict blood pressure testing was used through systematic training. For example, an unstable and inaccurate wrist sphygmomanometer was abandoned, and an upper arm inflatable sphygmomanometer was used in a unified way. Moreover, those who found high blood pressure must continuously measure it three times in a quiet state. Furthermore, these comprehensive measures were fully implemented after more than one year of training, so only the control rate data in 2017 are the closest to the real data, and the trend changes after that are more meaningful.

### 3.5. “Three Rates” Parameters for the Prevention and Treatment of Hypertension

The hypertension awareness rate and treatment rate increased annually during the WPPHCPP implementation and peaked in 2017 at 95.0% and 94.9%, respectively. The highest rate of disease control was 45.6% in 2016 (Table 6).

### 3.6. Changes in the Risk Classes in Hypertensive Patients

In the population who underwent the physical examinations, we selectively analyzed the records from the first and the last physical examination of patients with at least two physical examinations. Based on the blood pressure and risk factor data from the last physical examination, 26,982 people were included in the risk assessment classifications; this number was significantly lower than the 33,064 people included in the first physical examination. The numbers of low-, intermediate-, and high-risk people were all reduced to varying degrees. The percentage of high-risk individuals (24.6%) in the last physical examination was lower than that in the first physical examination (27.4%). The detailed classification changes are shown in Table 7.

### 3.7. Distribution of Factors Affecting the Cardiovascular Prognosis in Hypertensive Patients

Comparisons of risk factor exposures between the first and last physical examinations in adult hypertensive patients suggested that the percentage of the population who smoked cigarettes and consumed alcohol decreased from 30.6% to 27.2% and 25.1% to 22.0%, respectively. The percentage of the population with the TC level <5.7 mmol/L and LDL-C level >3.3 mmol/L decreased from 22.3% to 22.0% and 24.1% to 20.6%, respectively. The percentage of the population with obesity did not significantly change, while other parameters increased by varying degrees (Table 8).

### 3.8. Deaths Caused by Hypertensive Cardiovascular or Cerebrovascular Diseases

#### 3.8.1. Deaths Caused by Hypertensive Cardiovascular or Cerebrovascular Diseases in the Total Population

By analyzing the 2014–2017 data from the death monitoring system of Xuyong County, we found out that the number and rate of deaths from hypertensive cardiovascular or cerebrovascular diseases have declined annually since 2014. The annual growth also decreased and reached its lowest value of 275 (39.55/100,000) deaths in 2017. The annual growth rate of coronary heart diseases, cerebral infarction, and stroke also continued to decrease (Table 9).

#### 3.8.2. Deaths due to Hypertensive Cardiovascular or Cerebrovascular Diseases in Individuals

The analysis of the 2014–2017 data in the death monitoring system of Xuyong County indicated that beginning from 2014, the number of people who died of hypertensive cardiovascular and cerebrovascular diseases decreased annually, which in turn decreased the mortality rate. The annual growth also decreased and reached the lowest number of 71 (11.38/100,000) deaths in 2017. Furthermore, the annual growth of coronary heart disease, myocardial infarction, and cerebral infarction decreased (Table 10).

## 4. Discussion

Since October 2014, 452,200 permanent residents in Luzhou City of Xuyong County—a national state-level, poverty-stricken county—have been offered free physical examinations through the WPPHCPP. A total of 285,458 people underwent the physical examinations, accounting for >90% of the population aged >18 years. Based on the results of the physical examination, a management and intervention program for hypertensive patients involving the joint actions of 3 administrative levels of government agencies (village, town, and county) was implemented for approximately 3 years. The results of our study showed that the female population in Xuyong County of Luzhou City is slightly higher than the male population. There were also more middle-aged and elderly people in the population, which may be associated with the large numbers of male migrant workers leaving their hometown to work elsewhere [28]. The prevalence of hypertension was 18.5% among adults in the area, and this prevalence is different from that in the other sampled regions. The total number of people receiving physical examinations in the county was 285,458 compared to 451,755 in the whole country; this number was obtained in the most recent nationwide survey of people undergoing physical examinations. In this study, with the support of the government project and funds, all residents in the area were required by the government-led policy to join the free medical examination program, and the result was that >90% of the residents aged >18 years were included. Therefore, this study should have no evident selection bias, and the prevalence of adult hypertension in the study population can represent the target population as a whole. The prevalence of hypertension is lower than the national number reported by the state (27.9%) [13] and the prevalence rate in rural areas (22.9%) [29]. This outcome could be due to many young and middle-aged people leaving the area for migrant work and can serve as an important referential statistic for future similar surveys.

Recently, the WPPHCPP has achieved notable success in hypertension management, which is reflected in the following aspects: (1) The newly diagnosed hypertensive patients in the past 3 years accounted for 29.6% of the total number of local hypertensive patients, indicating that nearly 1/3 of the patients received the benefits of early detection and early diagnosis under the expanded scope of free physical examinations, which provided patients with the necessary conditions for early treatment and effective health management. (2) The number of patients with low, intermediate, and high risk of hypertension decreased by varying degrees, specifically high-risk patients, which decreased from 9062 (27.4%) to 6,640 (24.6%). This decrease is critical in reducing premature deaths caused by hypertension, effectively protecting the labor force. The cause of death analysis of the death monitoring system data also suggests that the number and rates of deaths related to hypertensive cardiovascular and cerebrovascular diseases had decreased annually, further confirming positive management outcomes. (3) The outcome parameters for health management of hypertensive patients and the “three rates” for disease prevention and control in the managed population showed continual improvements during the management and intervention process. The management rate was maintained above 80%, which is higher than the 55% rate in one of the urban regions of Luzhou City [30]. The rates of awareness, treatment, and control of hypertension were all higher than those at the national levels [31] (95.0% vs. 42.6%, 94.9% vs. 34.1%, and 40.3% vs. 9.3%, respectively) and higher than those in other rural areas of China [32]. (4) Lifestyle and risk factors affecting the prognosis of hypertensive patients exhibited significant improvements at various degrees. The percentage of the population with smoking habit, alcohol-drinking habit, TC level <5.7 mmol/L, and LDL-C level >3.3 mmol/L was all reduced. Other risk factors did not significantly change possibly due to the shorter intervention period.

The reasons for the good WPPHCPP results are unique and worth learning. The four major characteristics of the WPPHCPP are as follows: (1) Government leadership: government-led, leadership-oriented implementation is the core for the prevention and control of chronic diseases. From an administrative point of view, adhering to the “first-hand responsibility system” is essential for ensuring the smooth implementation of disease interventions [33]. The WPPHCPP implementation took full advantages of “leadership, management, supervision, and safeguarding” responsibilities of government agencies. In Xuyong County, a working group was established for the prevention and healthcare management, and the county party committee secretary and head of the county were the group leaders. An implementation plan was formulated with fixed-point contacts and responsibility assignments; additionally, a support system was formed through active collaborations between multiple departments, including the propaganda department of the county party committee, the county finance bureau, the county education bureau, and the bureau of culture, sports, radio, and television. The office of disciplinary inspections and supervision strengthened its supervision responsibility by incorporating WPPHCPP implementation into the government's objective assessments of townships and departments, aiming to improve the promotional efforts of the county, town, and village offices. A primary system of organization, mobilization, and management was established and supported by “government planning and coordinating, each department taking the responsibility of a specific aspect, township organizing and implementing, and residents actively participating.” The township health centers and village health stations were constructed and standardized and were provided with essential equipment such as automatic biochemical analyzers. Physical examinations, management funds, pensions, and insurance premium purchases for village doctors were all included in the budget to ensure the complete fulfillment of the government's responsibilities of “leadership, management, supervision, and safeguard” in the prevention and control of chronic diseases. (2) Multidepartment collaborations: the service model of the “medical community” in combination with the “joint health management system” established among the 3 levels of medical institutions of the county, township, and village has allowed quality medical resources to reach lower ranks of medical facilities. It also solved the problem of insufficient service capacities at the grassroots level, home of the foremost battlefield of chronic disease prevention and control, yet the primary medical services were not trusted by the residents due to their poor capacities [34]. To improve the effectiveness of the work, the County Health Planning Bureau established a county-level treatment and prevention expert team to formulate a technical WPPHCPP program to train the grassroots health service personnel in a centralized manner. A county-level medical system focusing on incorporating technologies was established through an entrusted management approach with integrated involvement of the county-level internal medical community, and county-level experts directly participated in village health management. The targeted trainings following a “teacher training students while practicing” motto allowed the experts to provide actual guidance to the grassroots health service personnel while conducting health management and having face-to-face live interactions with residents. The organically integrated approach of collectively training medical staff on-site has solved several existing issues faced by doctors in rural areas, including inefficient scheduling of daily trainings, scheduling conflicts in working and learning, impractical training content, and poor training outcomes [35]. The establishment of such a vertically integrated network of health services has significantly enhanced health service capacity in rural areas [36]. (3) Participation of the whole society, responsibility sharing, and management: the precise management with the “2 + 1” (home township health team, village doctors, and village leaders) face-to-face approach takes full advantage of the following facts: the villagers trust their leaders, doctors understand the local individuals' health, and county-level experts have service ability. It also used the “individual health intensive intervention plan” approach for specific populations, such as intensive face-to-face interventions for poor lifestyle habits and teaching disease prevention skills to improve the self-management abilities of hypertensive patients. This teamwork model has improved the satisfaction levels of rural residents with local health services [37], and the willingness and enthusiasm of the residents to actively participate in the WPPHCPP significantly increased. Simultaneously, maximally mobilizing the strength of “villages and communities” under the representations by their leaders increased the utilization rate of health resources in rural regions [38]. It also resulted in successful development of various forms of health education and health promotion activities that allowed more residents to take the initiative to participate in the implementation of practicing “group defense, group management” in chronic disease prevention and control [39]. (4) Specific mechanisms and intervention programs to fully support the implementation of the above strategies: first, by utilizing the government's special financial funds to provide free physical examinations for the entire population, we were able to screen for hypertensive patients, establish health records and health assessments, and provide stratified intervention and management of risk factors for high-risk hypertensive patients including treatment with prescription drugs, elimination of risk factors, and different forms of health education. Second, the joint actions of three levels of rural medical institutions supported by county-level experts establishing special clinics for hypertensive patients once every half-month helped the face-to-face teaching of health education knowledge, skills, and treatment options to grassroots doctors. Finally, fully utilizing the strength of the local village and community leaders, the WPPHCPP program provided bimonthly health workshops and testimonial seminars locally to hypertensive patients, which were organized by officials and doctors in the village. At these seminars, patients communicate with each other, present their own process of hypertension management, and share their current status and control of hypertension-related parameters. The on-site medical staff provides summaries of the experiences in successful management cases. They also emphasize incorrect viewpoints and practices while teaching self-management skills that are necessary to improve the self-management abilities of hypertensive patients.

The above results and experiences demonstrated that the comprehensive hypertension management and intervention model achieved by implementing the WPPHCPP in Xuyong County of Luzhou has met the objective of the latest summit held by the United Nations (UN) on the prevention and control of chronic diseases and should become a widely adapted model. Currently, a large gap still exists between the quality of medical service supplies and the expectations of the general public in many rural areas of China [39]. Derived from the NBPHS, the WPPHCPP in Xuyong County significantly accelerated the process of improving rural area preventive healthcare services and enhanced the awareness of the local villagers in preventing and treating disease. The project reduced poverties due to illness or subsequent returns to poverty after illness, proving to be an effective and healthy program to help impoverished populations. In September 2018, the third UN High-Level Meeting on Non-Communicable Disease Prevention and Control was held in New York. The summit emphasized the importance of government responsibilities, mobilizations of departments and social groups, and the role of public health in disease prevention and control [40]. Our nation holds unparalleled institutional, mobilizational, and organizational advantages in the above aspects. How to implement these strategic directions and integrate the strategies and tactics has been a global challenge; thus, the theme of the summit was “Time to Deliver.” Xuyong County's implementation of the hypertension prevention and control program through the WPPHCPP provides the best practice and case study for the “Time to Deliver” theme of the UN Summit. Therefore, the WPPHCPP model of Xuyong County is considered a significant reference worldwide, and it merits broader nationwide adaptation and practice, specifically in state-level poverty-stricken counties. In the future, we will continue to evaluate the effectiveness of the WPPHCPP, explore long-term and sustainable mechanisms, and increase the levels of rewards and punishments for project personnel to gather national preventive healthcare support from all populations.

This study did not focus on the serious complications of hypertension, specifically atrial fibrillation, but the ongoing study has begun to focus on monitoring the occurrence of various complications of hypertension. Moreover, this is a long-term project. Some effects and indicators will change over time because of complex reasons. Therefore, some evidence and explanations need to be strengthened. Currently, we are conducting an initial assessment, and we believe that this preliminary study will provide a summary and guidance for a more comprehensive assessment in the next study and better implementation of interventions for this project.

## 5. Conclusion

The implementation of the WPPHCPP in Xuyong County and its effective outcomes represent an example of meeting the latest UN's advocacy of the prevention and control of chronic diseases. It provides a global reference for the importance of proper healthcare management and can be adapted and practiced nationwide, particularly in poverty-stricken areas.

## Figures and Tables

**Table 1:** Risk classification of hypertensive patients.

Risk factors	Blood pressure		
	Grade 1 hypertension (SBP 140–159 or DBP 90–99)	Grade 2 hypertension (SBP 160–179 or DBP 100–109)	Grade 3 hypertension (SBP ≥180 or DBP ≤110)
No other risk factors	Low risk	Intermediate risk	High risk
1-2 risk factors	Intermediate risk	Intermediate risk	High risk
≥3 risk factors	High risk	High risk	High risk

**Table 2:** Social demographic distribution characteristics of the test population.

Factors	Number of persons	Composition (%, *N* = 285,458)
Gender		
Male	135,564	47.5
Female	149,827	52.5
Missing/unknown	67	0.0

Age (years)		
18–24	22,451	7.9
25–34	27,174	9.5
35–44	46,053	16.1
45–54	71,211	24.9
55–64	50,413	17.7
≥65	68,156	23.9

**Table 3:** Prevalence of hypertension in people aged >18 years (until 2017).

Factors	Number of patients	Prevalence, % (*N*)
All	50,813	18.5 (274,689)

Gender		
Male	24,528	18.7 (131,092)
Female	26,279	18.3 (143,597)

Age (years)		
18–24	12	0.1 (22,114)
25–34	168	0.6 (26,625)
35–44	2,376	5.4 (44,361)
45–54	10,296	15.2 (67,793)
55–64	12,138	25.3 (48,006)
≥65	25,823	39.2 (65,856)

**Table 4:** Prevalence of hypertension detected in people aged ≥18 years from 2015 to 2017 (*n* (%)).

Factors	2015	2016	2017	Subtotal
All	8,408 (16.5)	4,116 (8.1)	2,550 (5.0)	15,074 (29.6)

Gender				
Male	4,376 (17.8)	2,171 (8.8)	1,322 (5.4)	7,869 (32.1)
Female	4,029 (15.3)	1,945 (7.4)	1,228 (4.7)	7,202 (27.4)

Age (years)				
18–24	5 (45.5)	1 (9.1)	1 (9.1)	7 (63.6)
25–34	44 (26.8)	29 (17.7)	7 (4.3)	80 (48.8)
35–44	621 (26.2)	260 (11.0)	154 (6.5)	1,035 (43.6)
45–54	2,121 (20.6)	967 (9.4)	632 (6.1)	3,720 (36.1)
55–64	2,171 (17.9)	938 (7.7)	654 (5.4)	3,763 (30.9)
≥65	3,446 (13.4)	1,921 (7.4)	1,102 (4.3)	6,469 (25.0)

**Table 5:** Outcome parameters for hypertension health management in 2015–2017 (%).

Parameters	2015	2016	2017
Rate of management	81.7	87.1	88.4
Rate of standardized management	43.8	52.7	55.9
Rate of blood pressure control of the managed population	85.5	77.0	67.8

**Table 6:** “Three rates” population parameters for the prevention and treatment of hypertension in 2015–2017 (%).

Parameters	2015	2016	2017
Awareness rate	83.5	91.5	95.0
Treatment rate	93.7	93.9	94.9
Control rate of blood pressure	33.5	45.6	40.3

**Table 7:** Changes in risk classes in hypertensive patients (*n* (%)).

Risk class of hypertension	First physical examination	Last physical examination
Low risk	2,032 (6.1)	1,585 (5.8)
Intermediate risk	21,970 (66.4)	18,757 (69.5)
High risk	9,062 (27.4)	6,640 (24.6)
Total	33,064	26,982

Note: low risk: Grade 1 hypertension and no other risk factors; intermediate risk: Grade 1 hypertension and 1-2 risk factors, or Grade 2 hypertension and ≤2 risk factors; high risk: Grade 3 hypertension or ≥3 risk factors.

**Table 8:** Changes in the rates of risk factor exposures in hypertensive patients aged ≥18 years (*n* (%)).

Risk factors	First physical examination		Last physical examination	
	Number of people exposed	Exposure rate (%)	Number of people exposed	Exposure rate (%)
Cigarette smoking	12,243	30.6	10,891	27.2
Alcohol	10,015	25.1	8,801	22.0
Obesity (BMI ≥28 kg/m^2^)	5,857	14.7	5,761	14.7
Abdominal obesity (waist circumference: men ≥90 cm, women ≥85 cm)	10,896	27.3	10,738	27.5
Impaired glucose tolerance (fasting blood glucose 6.1–6.9 mmol/L)	3,486	9.4	3,584	11.9
Diabetes (fasting blood glucose ≥7.0 mmol/L)	3,862	10.4	4,327	14.3
Total cholesterol (TC ≥ 5.7 mmol/L)	7,962	22.3	6,373	22.0
LDL-C >3.3 mmol/L	8,067	24.1	5,870	20.6
HDL-C <1.0 mmol/L	3,269	9.8	4,798	16.8
Serum creatinine (men ≥133 *μ*mol/L, women ≥124 *μ*mol/L)	588	1.7	644	2.2

**Table 9:** Annual deaths of patients with hypertensive cardiovascular and cerebrovascular diseases in Xuyong County (*n* (1/100,000)).

Year	2014	2015		2016		2017	
	Number (rate) of deaths	Number (rate) of deaths	Annual growth (%)	Number (rate) of deaths	Annual growth (%)	Number (rate) of deaths	Annual growth (%)
Coronary heart diseases	145 (20.15)	279 (38.38)	90.51	362 (49.64)	29.31	351 (50.48)	1.71
Myocardial infarction	44 (6.11)	124 (17.06)	179.02	108 (14.81)	−13.20	146 (21.00)	41.80
Cerebral infarction	42 (5.84)	98 (13.48)	131.02	225 (30.85)	128.82	265 (38.11)	23.54
Stroke	5 (0.69)	28 (3.85)	454.45	69 (9.46)	145.60	53 (7.62)	−19.43

**Table 10:** Hypertensive cardiovascular and cerebrovascular diseases in individuals aged <70 years in Xuyong County (*n* (1/100,000)).

Year	2014	2015		2016		2017	
	Number (rate) of deaths	Number (rate) of deaths	Annual growth (%)	Number (rate) of deaths	Annual growth (%)	Number (rate) of deaths	Annual growth (%)
Coronary heart diseases	43 (6.43)	80 (11.85)	84.20	115 (17.00)	43.49	107 (17.15)	0.89
Myocardial infarction	15 (2.44)	38 (5.63)	150.83	53 (7.84)	39.22	65 (10.42)	32.98
Cerebral infarction	10 (1.50)	29 (4.30)	187.13	62 (9.17)	113.40	62 (9.94)	8.43
Stroke	0 (0.00)	7 (1.04)	—	20 (2.96)	185.20	17 (2.73)	−7.83

## Data Availability

The data used to support the findings of this study are included within the article.
